# Voice as a sensitive biomarker for predicting exercise intensity: a modelling study

**DOI:** 10.3389/fphys.2025.1483828

**Published:** 2025-04-28

**Authors:** Shuyi Zhou, Ruisi Ma, Wangjing Hu, Dandan Zhang, Rui Hu, Shengwei Zou, Dingyi Cai, Zikang Jiang, Hexiao Ding, Ting Liu

**Affiliations:** ^1^ School of Physical Education, Jinan University, Guangzhou, Guangdong, China; ^2^ The Third Affiliated Hospital, Sun Yat-Sen University, Guangzhou, Guangdong, China; ^3^ School of Nursing, Sun Yat-Sen University, Guangzhou, Guangdong, China; ^4^ School of Nursing, Jiangxi Medical College, Nanchang University, Nanchang, Jiangxi, China

**Keywords:** speech analysis, voice, exercise intensity, non-invasive monitoring, health assessment

## Abstract

**Objective:**

This study investigates the potential of using voice as a sensitive omics marker to predict exercise intensity.

**Methods:**

Ninety-two healthy university students aged 18–25 participated in this cross-sectional study, engaging in physical activities of varying intensities, including the Canadian Agility and Movement Skill Assessment (CAMSA), the Plank test, and the Progressive Aerobic Cardiovascular Endurance Run (PACER). Speech data were collected before, during, and after these activities using professional recording equipment. Acoustic features were extracted using the openSMILE toolkit, focusing on the Geneva Minimalistic Acoustic Parameter Set (GeMAPS) and the Computational Paralinguistics Challenge (ComParE) feature sets. These features were analyzed using statistical models, including support vector machine (SVM), to classify exercise intensity.

**Results:**

Significant variations in speech characteristics, such as speech duration, fundamental frequency (F0), and pause times, were observed across different exercise intensities, with the models achieving high accuracy in distinguishing between exercise states.

**Conclusion:**

These findings suggest that speech analysis can provide a non-invasive, real-time method for monitoring exercise intensity. The study’s implications extend to personalized exercise prescriptions, chronic disease management, and the integration of speech analysis into routine health assessments. This approach promotes better exercise adherence and overall health outcomes, highlighting the potential for innovative health monitoring techniques.

## 1 Introduction

Speech is a fundamental aspect of human communication, conveying not only linguistic information but also paralinguistic cues such as gender, age, emotional state, and health conditions (Docio-Fernandez et al., 2015; Lima et al., 2013; Zaman et al, 2021). The interaction between speech production and physiological states, including exercise, has been a subject of increasing interest. During physical activity, changes in respiratory patterns and subglottal pressure affect voice characteristics, making it feasible to monitor exercise intensity through speech analysis (Conrad and Nle, 1979; Rochet-Capellan and Fuchs, 2013; Trouvain and Truong, 2015; Dehak et al., 2011; Mohammadi et al., 2010). Despite the potential applications, there is a significant gap in knowledge regarding the use of speech as a biomarker for exercise intensity, particularly in non-English languages such as Mandarin.

The pursuit of voice biomarkers for exercise intensity is critical for several reasons. Traditional methods of assessing exercise intensity, such as heart rate monitoring and perceived exertion scales, have limitations. Heart rate monitors require physical contact and may not always be practical, while perceived exertion is subjective and can vary between individuals (Docio-Fernandez et al., 2015; Lima et al., 2013). A non-invasive, real-time monitoring method using voice analysis could provide a more practical and accessible alternative. Voice biomarkers could be especially valuable in settings where traditional monitoring equipment is unavailable or impractical.

Research has demonstrated that physical activity influences speech production. Physical activity refers to any physical movement produced by skeletal muscles that requires energy expenditure (World Health Organization, 2024). Variations in fundamental frequency (F0), speech duration, and pause times have been observed during different physical activities (Trouvain and Truong, 2015; Dehak et al., 2011; Mohammadi et al., 2010). These changes are attributed to physiological factors such as increased respiratory rate and subglottal pressure during exercise (Truong et al., 2015). For example, during high-intensity exercise, the vocal cords experience greater tension, resulting in higher F0. Similarly, increased physical exertion can lead to longer pauses in speech as the body demands more oxygen (Truong et al., 2015; Godin and Hansen, 2008; Godin and Hansen, 2011; Johannes et al., 2007). However, most existing research focuses on English speakers, and there is limited understanding of how these findings apply to Mandarin, the world’s most widely spoken language.

This study builds on the premise that speech production is closely linked to respiratory function (Schuller et al., 2014), which is directly affected by physical activity (Godin and Hansen, 2011). Understanding how exercise-induced changes in respiratory physiology, such as increased breathing rate and altered subglottal pressure, manifest in speech production forms the theoretical foundation of this research (Longmuir et al., 2018; Karvonen and Vuorimaa, 1988; Eyben et al., 2010; Zee, 1991). By analyzing speech features before, during, and after physical activities of varying intensities, the study aims to establish the feasibility of using voice as a non-invasive biomarker for exercise monitoring (Law et al., 2012). On top of this, the objective of this study is to determine whether speech analysis can reliably indicate exercise intensity. Specific objectives include identifying key speech features that change with exercise intensity, comparing these features across different exercise intensities (Longmuir et al., 2017; Schellenberg et al., 2007; Scott et al., 2013), and establishing statistical models to predict exercise intensity based on speech characteristics. The potential applications of this research are vast. For instance, speech analysis could be integrated into wearable health devices, providing continuous, non-invasive monitoring of exercise intensity. This could enhance personalized exercise prescriptions, improve chronic disease management, and support routine health assessments. By offering a practical and accessible monitoring method, this approach could promote better exercise adherence and overall health outcomes.

## 2 Materials and methods

### 2.1 Study design

This study adhered to the Transparent Reporting of a Multivariable Prediction Model for Individual Prognosis or Diagnosis (TRIPOD) guidelines (Supplementary Materia1 1 *-TRIPOD checklist*). It was a modelling study conducted using the speech data from a cross-sectional study. The study took place at the outdoor sports field of Jinan University from November 2023 to April 2024 and received approval from the Institutional Review Board (IRB) of Jinan University (Approval No. JNUKY-2023-0154). All student assistants involved in this study were thoroughly trained in advance to conduct the tests and operate the equipment proficiently.

In this study, we intended to figure out the relationship between exercise intensity and speech characteristics in a university population. Demographic and physical activity data were collected for all participants. Each participant wore a heart rate monitor throughout the testing period. Initially, participants recorded their speech and resting heart rate before beginning the Canadian Assessment of Physical Literacy-2 (CAPL2) physical competence test. Participants also performed a speech at the end of each exercise session, during which trained student assistants recorded their current heart rate and speech data. The average heart rate during speaking was used as an objective indicator of exercise intensity (Karvonen and Vuorimaa, 1988).

### 2.2 Participants

Ninety-Two healthy university students (48 males and 44 females) aged between 18 and 25 were recruited for this study. To maintain homogeneity of the sample, strict inclusion and exclusion criteria were implemented. All participants were fluent in Mandarin and had no history of speech disorders, cardiopulmonary diseases, or any other medical conditions that could affect their performance during the exercise tests. Prior to participation, each subject provided informed consent and underwent a comprehensive physical examination to ensure their suitability for the study. The inclusion criteria were standardized to mitigate potential biases, requiring all participants to have at least 6 months of regular physical activity experience. Exclusion criteria included a history of voice impairment, neuromuscular disorders affecting speech and/or breathing for speech, and any other health conditions that could confound the voice outcome measures. These criteria ensure the homogeneity of the sample and the reliability of the study results.

### 2.3 Language and reading material

Mandarin, also known as Putonghua, is the most widely spoken language globally, with over a billion native speakers. It serves as the official language of China and one of the official languages of Singapore, facilitating communication across diverse linguistic groups within these regions. However, Mandarin is understudied in the context of exercise speech analysis. Therefore, this study focuses on Mandarin to fill this research gap. By examining how Mandarin speech features change with varying exercise intensities, we aim to contribute to the broader understanding of exercise physiology and linguistics in a language that has not been extensively researched in this domain. This focus on Mandarin not only broadens the scope of existing research but also provides valuable insights that are culturally and linguistically relevant to a significant portion of the global population.

Participants were asked to read a short text aloud in Mandarin (Supplementary eFigure 1). The text comprised two parts: the first part was the well-known speech study text “The North Wind and the Sun” (Mandarin version). The second part consisted of five long vowel characters, each of which participants were asked to pronounce for approximately 3 s and then repeat.

### 2.4 Research data

In this study, we used openSMILE 3.0, an open-source toolkit for speech signal processing, to extract speech features (Eyben et al., 2010). This toolkit extracts acoustic parameters that describe paralinguistic characteristics of speech. Speech data were collected before, during, and after the physical activities, recorded by trained assistants. These recordings were segmented and analyzed to extract relevant features, which were subsequently used to train classification models to predict exercise intensity. The dataset was split into training, validation, and test sets. The training set was used to train the models, the validation set was used to tune the model parameters, and the test set was used to evaluate the final model performance.

The speech data was recorded using a professional recorder (DR-44WL, TASCAM Ltd., CHINA), which allowed for amplification of the input signal and simultaneous recording of separate audio channels. The recorder was positioned approximately 30 cm in front of the speaker’s mouth. Inevitably, light ambient noises such as walking and talking sounds were included. The gain of the recorder was maximized to keep the noise level below −30dB, and the sampling rate was set at 44.1 kHz. The assistant responsible for collecting speech data underwent professional training to become proficient in using the equipment.

### 2.5 Features of voice to analysis

To analyze the speech data, we used two well-known sets of features: the Geneva Minimalistic Acoustic Parameter Set (GeMAPS) and the Computational Paralinguistics Challenge (ComParE) (Schuller et al., 2016; Eyben et al., 2016). These feature sets help us understand different aspects of speech and have been used successfully in various studies to assess personality, detect speech-related diseases (Xue et al., 2019), and identify gender and age (Schuller et al., 2013).

GeMAPS includes 88 specific measurements that describe various characteristics of speech sounds. These features cover frequency-related aspects such as the fundamental frequency (F0) and formant frequencies, energy-related aspects including shimmer and loudness, and temporal aspects like the rate of loudness peaks. These features are known to reflect emotional properties in speech (Eyben et al., 2016).

ComParE is a much larger set of features, including 6,373 different measurements. These features are derived from energy-related low-level descriptors (LLDs), spectral LLDs, sound-related LLDs, and various functionals applied to these descriptors. ComParE has been used to analyze cognitive load, physical load, emotion, and speech-related diseases. It provides a detailed and comprehensive description of speech sounds (Schuller et al., 2016; Eyben et al., 2016).

### 2.6 Outcomes

The primary outcome of this study was to assess exercise intensity and its impact on speech features. We utilized three primary physical activities from CAPL-2 protocol to evaluate exercise intensity: the PACER test, the CAMSA, and the Plank test. Additionally, we collected speech data during rest as a baseline.

Participants performed the PACER (Progressive Aerobic Cardiovascular Endurance Run), a high-intensity activity where they ran back and forth across a 20-m space at increasing speeds, following audio cues (Leger et al., 1988). The test continued until the participant could no longer keep up with the pace. This exercise significantly elevated heart rate and measured cardiovascular endurance, providing valuable data on how intense physical exertion affects speech features. These insights can aid in developing exercise prescriptions aimed at improving cardiovascular health.

The Canadian Agility and Movement Skill Assessment (CAMSA) involved moderate-intensity tasks such as running, jumping, and balancing (Longmuir et al., 2017). Participants completed a timed obstacle course that tested their motor skills and coordination. This activity enhanced overall fitness and agility, offering a balanced exercise regimen that supports cardiovascular health and functional fitness, which are crucial for various populations, including those recovering from illnesses.

The Plank test required participants to hold a plank position, maintaining a straight line from head to heels, for as long as possible (Bohannon et al., 2018). This moderate-intensity exercise assessed core muscle strength and endurance. The data collected from this test helped design exercise programs that focus on core stability and muscular endurance, essential for maintaining overall physical fitness.

Heart rate measurements were continuously monitored during each of these exercises using Fitbit wristband, which served as a benchmark for validating the speech features as indicators of exercise intensity. Speech data were also collected during periods of rest to serve as a baseline for comparison with the speech data collected during physical activities. This baseline was crucial for understanding the impact of different exercise intensities on speech characteristics.

### 2.7 Statistical analysis

In this study, we utilized the scikit-learn 1.0.1 package, a widely-used library in Python for machine learning (Pedregosa et al., 2011). Scikit-learn provides a range of efficient tools for data analysis and modeling, including classification, regression, clustering, and dimensionality reduction. Specifically, we employed scikit-learn’s support vector machine (SVM) implementation to classify exercise intensity based on speech features. The SVM algorithm was chosen for its robustness in handling high-dimensional data, making it well-suited for the complex and varied speech features analyzed in this study.

Descriptive statistics were employed to describe the participants’ characteristics. Before conducting the main analyses, the assumptions of normality, homoscedasticity, and linearity were examined and confirmed. A one-way Repeated Measures Analysis of Variance (RM-ANOVA) was used to investigate differences in speech features across the different exercise states. Pearson’s product-moment correlation and simple linear regression were performed to examine the relationships among each speech feature.

Following this exploratory analysis, we implemented SVM to automatically classify each exercise intensity. To enable short-time processing, we split the speech data into segments. The audio data were segmented from 0 to 10 s, then the next 10-s segment was extracted with no overlap, and so forth. The final part, which was shorter than 10 s, was discarded. All speech data were randomly divided into training, development, and test datasets in a ratio of 6:2:2. The training dataset was used to fit the model, the development dataset provided an unbiased evaluation of the model fit while tuning hyperparameters, and the test dataset was used for the final model evaluation. By combining exploratory statistical analysis with automated classification, we aimed to robustly assess the differences in speech features across exercise states and to classify physical load based on speech features.

**FIGURE 1:**
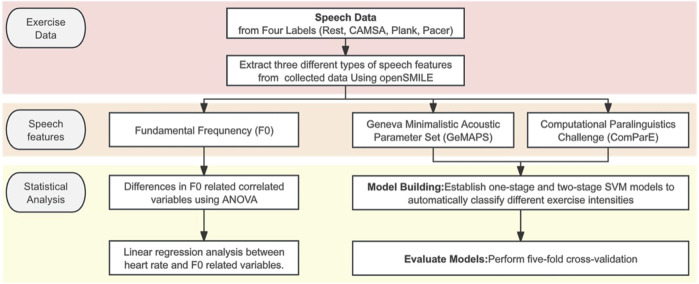
Flowchart of research process.

The second experiment involved a two-stage approach: first, a three-class SVM model classified rest, moderate-intensity, and high-intensity exercises; second, a two-class SVM model classified CAMSA and plank test. To ensure reliable results, five-fold cross-validation was used for the three-class SVM. In each fold, the data were balanced to ensure an even distribution of male and female participants. This cross-validation method helped mitigate any potential bias introduced by gender imbalance in the sample. After training with all mixed audio samples, the effects of training with separate corpora were examined. Unweighted average recall (UAR), precision, and F1-scores assessed classification accuracy. Figure 1 summarizes the entire research process.

## 3 Results

A total of 92 native speakers (48 males and 44 females) participated in the study, with ages ranging from 18 to 22 years (Overall: M = 19.01, SD = 0.93; Males: M = 19.11, SD = 0.95; Females: M = 18.83, SD = 0.85). Prior to using RM-ANOVA, skewness and kurtosis for all variables, including gender categories, were tested and found within ±1.96. Shapiro-Wilk tests were non-significant for all scores, confirming normality. The Fmax coefficient for speech duration was less than ten (Fmax = 1.92), meeting the assumption of homogeneity of variance. Mauchly’s W was 0.402 and significant (p < 0.001), indicating a violation of sphericity, so the Greenhouse-Geisser correction was applied.

### 3.1 Analysis of speech duration and fundamental frequency (F0)

Before the experiments, we first selected five speech variables—(a): Mean speech duration, (b): Mean F0, (c): F0 range, (d): Pause times, (e): Pause duration—as indicators of exercise intensity. We conducted a preliminary analysis of these variables to explore the potential impact of exercise intensity on speech characteristics.

**FIGURE 2:**
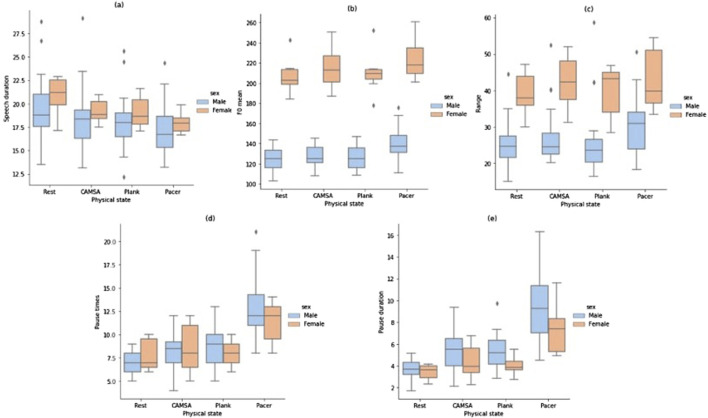
Comparison of speech features under different exercise intensities.

RM-ANOVA revealed statistically significant differences in speech duration means (F = 7.89, p = 0.001, η^2^ = 0.21). Pairwise comparisons with Bonferroni adjustments showed that speech duration in the rest state (M = 19.82, SD = 3.21) was significantly longer than in the pacer state (M = 17.35, SD = 2.32), p = 0.003, 95% CI [0.68, 4.26], d = 0.70. Pacer speech duration was also significantly shorter than in the CAMSA state (M = 18.56, SD = 3.13), p = 0.014, 95% CI [−2.26, −0.18], d = 0.60, and the plank state (M = 18.23, SD = 2.74), p = 0.049, 95% CI [−1.76, −0.01], d = 0.51. No significant differences in speech duration were found among the other three movement states (Figure 2A).

The mean F0 in each state was examined using RM-ANOVA, with no violation of the homogeneity of variance assumption (Fmax = 1.14). Mauchly’s W was 0.52 and significant (p = 0.002), so the Greenhouse-Geisser correction was applied. The RM-ANOVA indicated significant differences in mean F0 (F = 34.32, p < 0.001, η^2^ = 0.53). Pairwise comparisons showed that the mean F0 in the pacer state (M = 158.11, SD = 39.89) was significantly higher than in the rest state (M = 143.55, SD = 37.36), p < 0.001, 95% CI [8.58, 20.53], d = 1.24; CAMSA state (M = 146.74, SD = 39.85), p < 0.001, 95% CI [6.26, 16.48], d = 1.13; and plank state (M = 144.53, SD = 39.02), p < 0.001, 95% CI [9.02, 18.13], d = 1.51. No significant differences were found among the other three states (Figure 2B).

The 75% and 25% intervals of F0 were used to assess the range of speech fluctuations in each state. Homogeneity of variance (Fmax = 1.58) and sphericity (p = 0.11) were supported. RM-ANOVA showed significant differences in F0 ranges across states [F (3, 90) = 10.68, p < 0.001, η^2^ = 0.26]. Pairwise comparisons with Bonferroni adjustments revealed that the F0 range in the pacer state (M = 33.47, SD = 9.45) was significantly larger than in the rest state (M = 28.48, SD = 8.49), p < 0.001, 95% CI [1.99, 7.99], d = 0.84, and the plank state (M = 28.93, SD = 10.68), p < 0.001, 95% CI [1.33, 7.75], d = 0.72, but not the CAMSA state. The F0 range in the CAMSA state (M = 30.74, SD = 9.97) was also significantly larger than in the rest state, p = 0.02, 95% CI [0.22, 4.31], d = 0.56 (Figure 2C).

Pause times were examined using one-way RM-ANOVA. Homogeneity of variance (Fmax = 4.11) and sphericity (p = 0.09) were supported. Results showed significant differences in pause times across states (F (3, 90) = 60.21, p < 0.001, η^2^ = 0.67). Pairwise comparisons with Bonferroni adjustments revealed that pause times in the pacer state (M = 12.55, SD = 2.92) were significantly greater than in the rest state (M = 7.16, SD = 1.44), p < 0.001, 95% CI [3.86, 9.92], d = 1.78; CAMSA state (M = 8.39, SD = 2.16), p < 0.001, 95% CI [2.87, 5.45], d = 1.64; and plank state (M = 8.65, SD = 1.99), p < 0.001, 95% CI [2.71, 5.10], d = 1.66. Pause times in the rest state were also significantly less than in the CAMSA state, p = 0.02, 95% CI [−2.29, −0.16], d = 0.59, and plank state, p = 0.01, 95% CI [−2.60, −0.37], d = 0.67. No significant differences were found between the CAMSA and plank states (Figure 2D).

Pause duration was also examined. Homogeneity of variance was not met (Fmax = 14.72), and sphericity was violated (p < 0.001). The Greenhouse-Geisser correction indicated significant differences in pause duration [F (3, 90) = 63.27, p < 0.001, η^2^ = 0.68]. Pairwise comparisons showed that pause duration in the pacer state (M = 9.01, SD = 3.13) was longer than in the rest state (M = 3.63, SD = 0.81), p < 0.001, 95% CI [3.78, 6.96], d = 1.71; CAMSA state (M = 5.08, SD = 1.84), p < 0.001, 95% CI [2.54, 5.32], d = 1.44; and plank state (M = 5.01, SD = 1.60), p < 0.001, 95% CI [2.69, 5.31], d = 1.54. Pause duration in the rest state was significantly shorter than in the CAMSA state, p < 0.001, 95% CI [−2.31, −0.58], d = 0.85, and the plank state, p < 0.001, 95% CI [−2.20, −0.54], d = 0.84. No significant differences were found between the CAMSA and plank states (Figure 2E).

### 3.2 Correlation analysis and regression modeling

The Pearson’s product-moment correlation coefficient (*r*) was computed to determine the magnitude and direction of the linear relationship between heart rate and various speech features (Table 1). Subsequently, standard linear regression was conducted to evaluate if heart rate significantly predicted speech features (Table 2). Given the significant differences in F0 values between male and female pronunciations (Eyben et al., 2016), separate regression analyses were performed for each gender. Significant correlations were found for most groups, and the majority of regression equations had an R^2^ above 20%, indicating that at least 20% of the variance in our speech data could be explained by heart rate. For males, heart rate predicted three features: 1) F0 mean = 110.90 + 0.16 * heart rate; 2) Pause times = 4.25 + 0.05 * heart rate; 3) Pause duration = 1.26 + 0.04 * heart rate. For females, heart rate predicted four features: 1) F0 mean = 164.13 + 0.42 * heart rate; 2) Range = 26.77 + 0.12 * heart rate; 3) Pause times = 4.43 + 0.04 * heart rate; 4) Pause duration = 0.57 + 0.04 * heart rate.

**TABLE 1:** Correlations between heart rate and speech features.

	Speech duration	F0 mean	Range	Pause times	Pause duration
Heart rate (All)	−0.22*	0.27**	0.30**	0.47**	0.45**
Heart rate (Male)	−0.24*	0.39**	0.24*	0.47**	0.46**
Heart rate (Female)	−0.26	0.66**	0.54**	0.51**	0.56**

Note: **Correlation is significant at the .01 level (2-tailed); *Correlation is significant at the .05 level (2-tailed).

**TABLE 2:** Results of the standard linear regression analysis between heart rate and speech features.

	*β (SE)*	*F (df)*	R^2^	△R^2^
Males				
Speech duration	−0.02 (0.01)	5.71 (1.94)	0.06	0.05
F0 mean	0.16 (0.04)	17.11 (1.94)	0.15	0.15
Range	0.06 (0.02)	5.78 (1.94)	0.06	0.05
Pause times	0.05 (0.01)	26.90 (1.94)	0.22	0.21
Pause duration	0.04 (0.01)	25.18 (1.94)	0.21	0.20
Females				
Speech duration	−0.02 (0.01)	1.91 (1.26)	0.07	0.03
F0 mean	0.42 (0.09)	20.49 (1.26)	0.44	0.42
Range	0.12 (0.04)	10.60 (1.26)	0.29	0.26
Pause times	0.04 (0.01)	9.03 (1.26)	0.26	0.23
Pause duration	0.04 (0.01)	11.60 (1.26)	0.31	0.28

Note: *β (SE)*, Regression Coefficient (Standard Error); *F (df)*, F-test value (Degrees of Freedom); R^2^, coefficient of determination; △R^2^, Change in R^2^ (Incremental R^2^).

### 3.3 Classification model performance

The Unweighted Average Recalls (UARs) of the best-performing classifiers using SVM are presented in Tables 3–5. We compared the classification performance of two feature sets: eGeMAPS and ComParE. Overall, the ComParE feature set outperformed eGeMAPS. The two-stage modeling approach (UAR_ComParE_ = 0.64) yielded better results than the one-stage modeling approach (UAR_ComParE_ = 0.58). A significant challenge was distinguishing between the two moderate-intensity exercises (CAMSA and plank test), as they exhibited highly similar speech features even in binary classification models.

**TABLE 3:** Results of four-class SVM using the eGeMAPS feature set.

	Rest	CAMSA	Plank	PACER	Precision	Recall	F1-score
Rest	**95**	24	36	6	0.62	0.60	0.61
CAMSA	24	**62**	9	33	0.50	0.44	0.47
Plank	36	9	**24**	3	0.33	0.32	0.32
PACER	6	0	3	**83**	0.68	0.93	0.79
Total					0.53	0.57	0.55

Note: CAMSA, canadian agility and movement skill assessment; PACER, progressive aerobic cardiovascular endurance run. Bold values indicate the number of correctly classified instances.

**TABLE 4:** Results of four-class SVM using the ComParE feature set.

	Rest	CAMSA	Plank	PACER	Precision	Recall	F1-score
Rest	**125**	21	12	3	0.64	0.77	0.70
CAMSA	39	**50**	21	33	0.38	0.38	0.38
Plank	51	39	**44**	3	0.50	0.34	0.41
PACER	3	24	3	**74**	0.66	0.71	0.68
Total					0.55	0.55	0.54

Note: CAMSA, canadian agility and movement skill assessment; PACER, progressive aerobic cardiovascular endurance run. Bold values indicate the number of correctly classified instances.

**TABLE 5:** Results of two-stage SVM.

	eGeMAPS (three-class SVM)			Recall	ComParE (three-class SVM)			Recall
	Rest	Moderate	High		Rest	Moderate	High	
Rest	**83**	71	6	0.51	**110**	47	3	0.67
Moderate	89	**151**	6	0.62	74	**155**	18	0.63
High	3	36	**86**	0.69	0	50	**74**	0.60
				0.61				0.63

	eGeMAPS (two-class SVM)			Recall	ComParE (two-class SVM)			Recall
	CAMSA	Plank			CAMSA	Plank		
CAMSA	**83**	45		0.65	**80**	47		0.63
Plank	65	**53**		0.45	39	**80**		0.68
				0.55				0.65
Total				0.58				0.64

Note: ComParE, computational paralinguistics challenge; CAMSA, canadian agility and movement skill assessment. Bold values indicate the number of correctly classified instances.

To ensure the robustness of our findings, a five-fold cross-validation was employed, which involves dividing the data into five subsets, training the model on four subsets, and testing it on the remaining subset. This process is repeated five times, with each subset used exactly once as a test set (Table 6). With additional training and testing data, both feature sets showed strong performance (UAR_eGeMAPS_ = 0.72, UAR_ComParE_ = 0.72). All models performed well in binary classification. The two-class model using the ComParE feature set achieved a UAR of 0.96 for distinguishing between rest and high-intensity exercise states, and a UAR of 0.79 for distinguishing between rest and moderate-intensity exercise states.

**TABLE 6:** Results of cross-validation.

	eGeMAPS (three-class SVM)			Recall	ComParE (three-class SVM)			Recall
	Rest	Moderate	High		Rest	Moderate	High	
Rest	**389**	336	27	0.65	**490**	244	18	0.66
Moderate	199	**942**	101	0.69	214	**920**	110	0.70
High	9	202	**525**	0.81	21	199	**517**	0.80
Total				0.72				0.72

Note: ComParE, computational paralinguistics challenge. Bold values indicate the number of correctly classified instances.

### 3.4 Validation of classification models across different corpora

Based on the results, the eGeMAPS framework was utilized to assess classification performance across various corpora with 3-s segments. Tables 7, 8 present the classification outcomes using corpora A and B, respectively. In the model based on Corpus A, classification accuracy significantly improved, effectively distinguishing between rest and PACER test exercise states. However, moderate-intensity exercise states, such as CAMSA and plank test states, remained challenging to differentiate. In contrast, corpora B, which consist of vowel, showed lower accuracy due to their smaller sample sizes.

**TABLE 7:** Results of four-class SVM using the eGeMAPS with Corpus A.

	Rest	CAMSA	Plank	PACER	Precision	Recall	F1-score
Rest	**92**	45	42	5	0.65	0.70	0.67
CAMSA	25	**100**	22	20	0.41	0.43	0.42
Plank	12	60	**85**	10	0.48	0.53	0.50
PACER	2	30	10	**146**	0.77	0.81	0.79
Total					0.58	0.61	0.64

Note: CAMSA, canadian agility and movement skill assessment; PACER, progressive aerobic cardiovascular endurance run. Bold values indicate the number of correctly classified instances.

**TABLE 8:** Results of four-class SVM using the eGeMAPS with Corpus B.

	Rest	CAMSA	Plank	PACER	Precision	Recall	F1-score
Rest	**28**	18	8	2	0.75	0.50	0.60
CAMSA	5	**38**	5	5	0.43	0.71	0.54
Plank	3	24	**26**	19	0.57	0.36	0.44
PACER	1	7	6	**38**	0.59	0.73	0.65
Total					0.58	0.57	0.55

Note: CAMSA, canadian agility and movement skill assessment; PACER, progressive aerobic cardiovascular endurance run. Bold values indicate the number of correctly classified instances.

## 4 Discussion

Consistent with previous studies on speech during exercise (Godin and Hansen, 2008), this study found that certain manually extracted features can reflect the intensity of exercise. The duration of pronunciation is affected by physical stress. Similar to prior studies (Godin and Hansen, 2008), this study found statistically significant differences in speech duration across different exercise intensities, with vigorous exercise having a more pronounced effect. The findings highlight the significance of analyzing speech characteristics in assessing physical fitness and tailoring exercise prescriptions. Further research is needed to refine these models and explore their applications in different populations and settings.

### 4.1 The exploration analysis of voice and exercise

F0, a widely studied parameter, varies with different conditions. This study analyzes its variation under various motion intensities and finds that F0 significantly increases under physical stress. Controlling vocal cords becomes challenging when fatigued, leading to increased F0 due to stronger vocal cord vibrations (Van Puyvelde et al., 2018; Godin and Hansen, 2015). Notably, F0 during vigorous exercise is higher than at moderate intensity or rest, but moderate intensity shows no significant change from the resting state, indicating maintained control over vocal cords. Both men and women show similar trends despite natural pitch differences.

The relationship between heart rate and speech features is critical in this context. As exercise intensity increases, the body undergoes physiological changes, most notably an increase in heart rate. These changes are mirrored in speech production, where factors such as subglottal pressure and respiratory patterns are influenced by the body’s need to meet the oxygen demands of physical exertion. For instance, the study observed that as heart rate increased, so did the pause times and pause durations in speech, reflecting the increased respiratory demand during more intense physical activity. These correlations suggest that heart rate can serve as a supportive measure alongside speech features to more accurately gauge exercise intensity.

The study also analyzes the range of F0 during exercise, showing that higher intensity exercises lead to unstable high and low intervals due to rapid, fast-paced breathing (Migliaccio et al., 2023). This instability results from overexertion or underexertion of articulation, causing pitch drops and sentence-breaking pauses (Niebuhr et al., 2020). Increased exercise intensity results in more frequent pauses, often occurring at punctuation or arbitrary points within sentences, due to the body’s need for more oxygen. The number of pauses is a significant parameter, effectively distinguishing between different exercise intensities but not between different types of exercises at the same intensity (Mahmod et al., 2022). Similarly, pause duration increases with exercise intensity, complementing the number of pauses as a useful parameter. While pause duration varies individually, it consistently differentiates exercise intensities (Hofmann and Tschakert, 2017). The study also explores using heart rate to predict speech features, finding a good linear correlation for the number and duration of pauses, although not for F0 or speech duration. This linear relationship between pauses and exercise intensity could enhance future research and machine learning models in this area.

### 4.2 Performance of classification models

While the four-classification results show only average results, the evaluation of two-stage model shows a satisfied result. The two feature sets, ComParE and eGeMAPS, yielded similar results overall. Both feature sets demonstrated strong performance, with the ComParE feature set achieving a slightly higher Unweighted Average Recall (UAR) in distinguishing rest from high-intensity exercise states. Specifically, ComParE had a UAR of 0.98, while eGeMAPS had a UAR of 0.94. These results underscore the effectiveness of both feature sets in capturing the distinct differences between resting and high-intensity exercise conditions. The high UAR values indicate that both feature sets are highly reliable for this classification task. In addition, the ComParE and eGeMAPS feature sets both showed commendable performance in distinguishing rest from moderate-intensity exercise states, though the results were not as high as those for high-intensity states. Specifically, the eGeMAPS feature set achieved a UAR of 0.64, while ComParE achieved a slightly higher UAR of 0.69. These results indicate that both feature sets are capable of differentiating between rest and moderate-intensity exercise conditions, with ComParE having a slight edge. The lower UARs compared to high-intensity differentiation suggest that distinguishing between rest and moderate-intensity exercise may be more challenging due to subtler differences in speech features at these intensity levels. Nevertheless, both feature sets provide reliable performance for exercise state classification, highlighting their utility in exercise monitoring applications. The models struggled to differentiate between moderate-intensity exercises like CAMSA and plank tests due to their similar speech features. Moderate-intensity exercises often have similar movement patterns and exertion levels, leading to comparable respiratory and vocal characteristics. The two-stage modeling approach showed better performance (UARComParE = 0.64) than the one-stage approach (UARComParE = 0.58), indicating that breaking down the classification task into sub-tasks enhances accuracy (Silla and Freitas, 2011). Validation with different corpora revealed varying levels of accuracy. The higher accuracy with Corpus A compared to Corpus B can be attributed to larger sample sizes and more varied data in Corpus A. Corpus B’s lower accuracy underscores the challenges posed by limited data and the importance of robust training datasets (Khoshgoftaar et al., 2013).

### 4.3 Implication of healthcare

This study’s findings hold significant implications for healthcare, particularly in the domains of exercise prescription and the management of chronic diseases. The ability to accurately classify exercise intensity using speech features introduces a novel, non-invasive method for monitoring physical activity. This can greatly benefit patients with cardiovascular diseases, as precise exercise intensity monitoring is crucial for tailored exercise prescriptions, reducing the risk of adverse events and enhancing cardiovascular health outcomes (Garber et al., 2011). Moreover, for patients with conditions like breast cancer, where fatigue and physical activity levels must be closely monitored, speech analysis offers a practical solution. Studies have demonstrated the benefits of personalized exercise regimens in improving patient quality of life and reducing cancer-related fatigue (Schmitz et al., 2010). The use of speech features to assess physical activity can thus support healthcare providers in delivering personalized and adaptive exercise plans, improving overall treatment efficacy. Additionally, incorporating speech analysis into routine health assessments can facilitate early detection of changes in physical fitness and health status. This aligns with the growing emphasis on preventive healthcare and the need for innovative tools to support health monitoring (Piwek et al., 2016). By providing a real-time, accessible measure of exercise intensity, this approach can promote more active lifestyles and better adherence to exercise recommendations, ultimately contributing to improved population health outcomes.

## 5 Limitations

Although the samples in this study were all tested for normality, the female sample was relatively short. Future studies could include more female data to ensure the reliability of the study findings. In addition, the modelling of this study used its own sample, which may affect the prediction model to some extent. With more data, some dependent variables with less predictive effects might have obtained better results. Third, the bad performance on Plank may be caused by the similar speech features under the same exercise intensity. Lastly, the openSMILE suite of programs was trained on Caucasian speakers, and therefore the constants used by openSMILE may not fully capture the speech patterns of Mandarin speakers. Future studies could explore using Mandarin-specific speech analysis tools or adjust openSMILE’s parameters to better suit the characteristics of Mandarin speech.

## 6 Conclusion

The results of this study suggest that speech analysis can provide a non-invasive, real-time method for monitoring exercise intensity. This approach promotes better exercise adherence and overall health outcomes, highlighting the potential for innovative health monitoring techniques. Additionally, by focusing on Mandarin, this research addresses a significant gap in the current literature. Future studies could expand on these findings by exploring other non-English languages to determine whether the speech markers identified here are universally applicable or if language-specific variations exist. Such research would contribute to a more comprehensive understanding of the intersection between language, speech, and physical exertion, ultimately leading to more personalized and effective health monitoring solutions.

## Data Availability

The raw data supporting the conclusions of this article will be made available by the authors, without undue reservation.
