# Supplementary material for: Voice as a sensitive biomarker for predicting exercise intensity: a modelling study
Source: Front Physiol. 2025 Apr 28;16:1483828. doi: 10.3389/fphys.2025.1483828 (PMC12066516; doi:10.3389/fphys.2025.1483828)

Supplementary Material

# Supplementary 1: TRIPOD guidelines

# Supplementary 2 eTable 1: Record of Canadian Agility and Movement Skill Assessment (CAMSA)


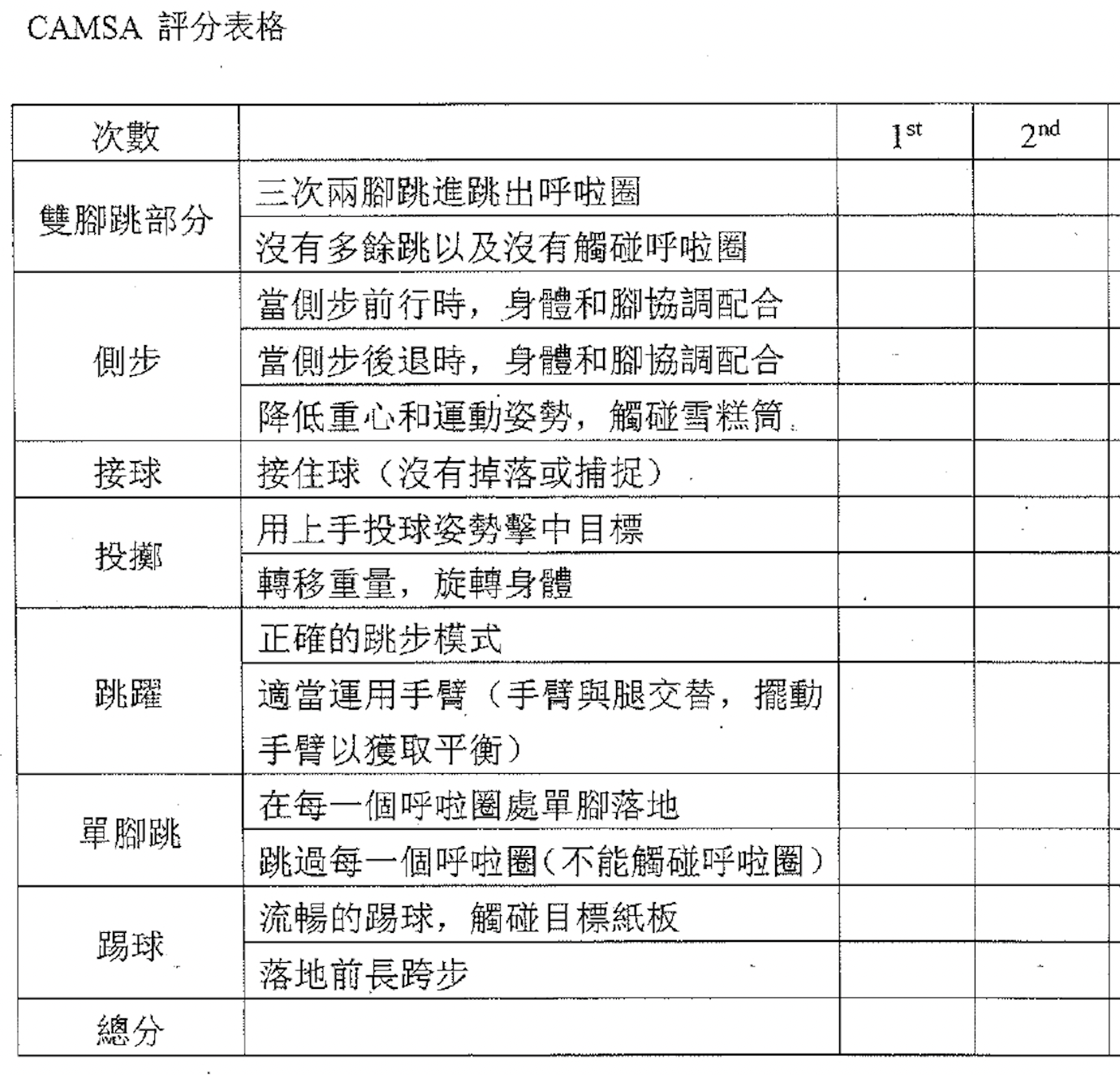


# Supplementary 2 eTable 2: Record of Progressive Aerobic Cardiovascular Endurance Run (PACER)


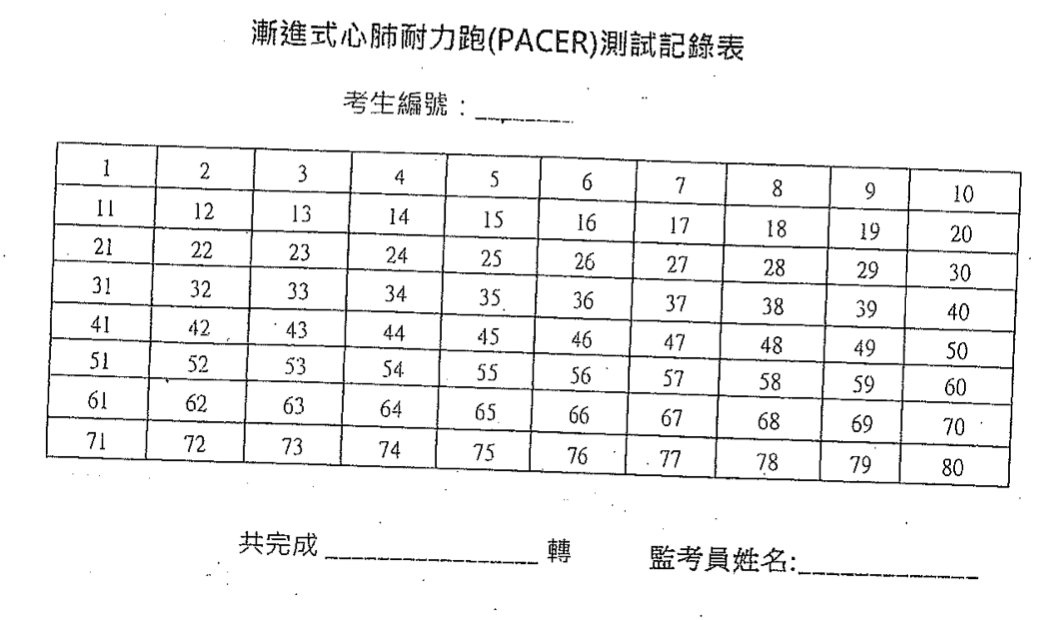


# Supplementary 2 eTable 2: Record of PLANK


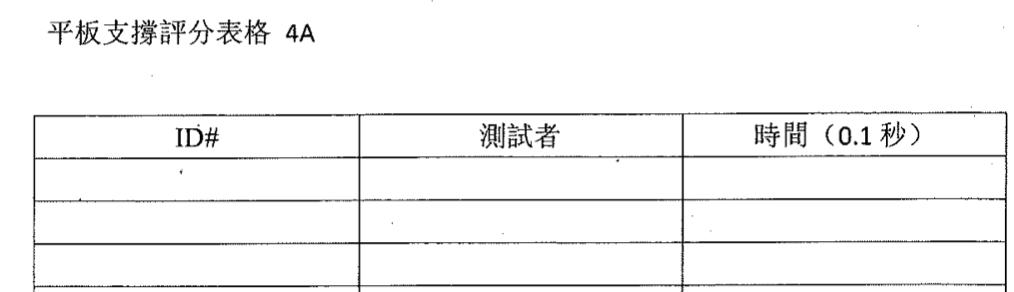


# Supplementary 2 eFigure 1: Reading materials (Mandarin version and English version)


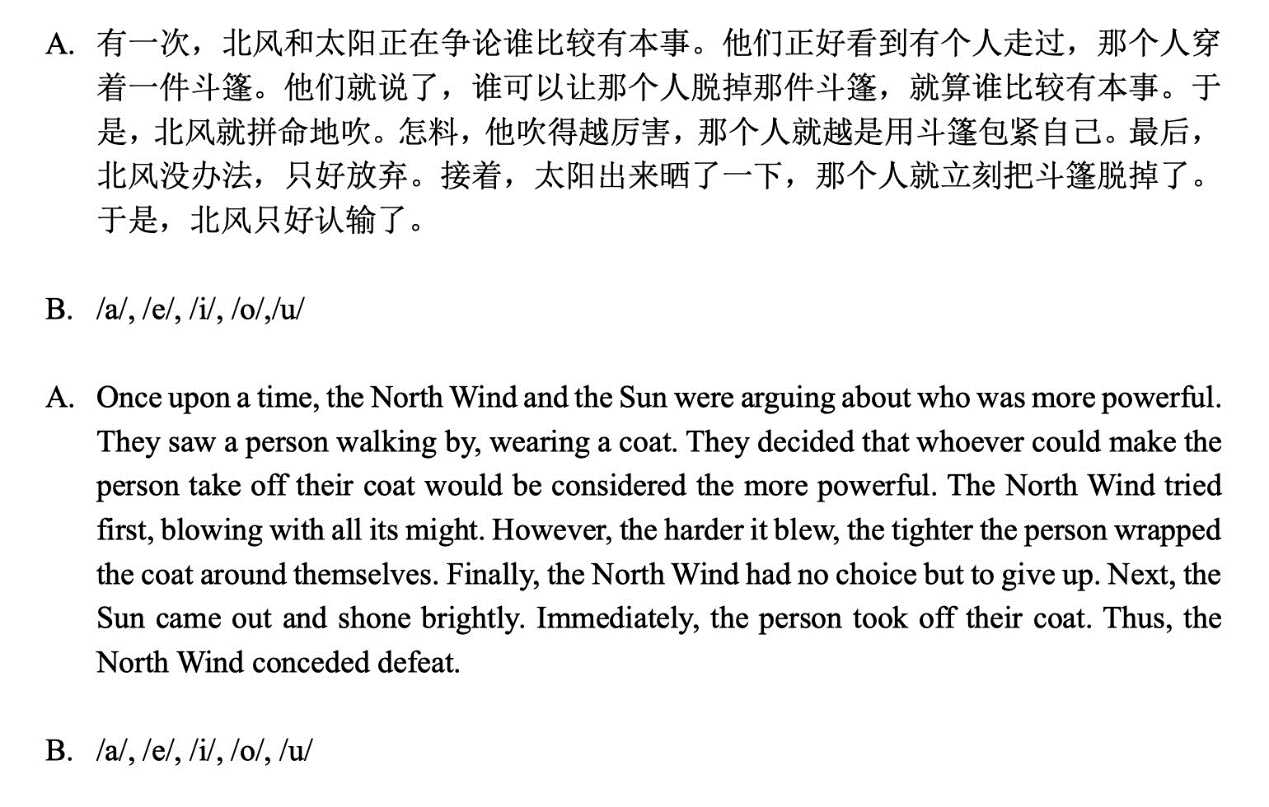


# Supplementary 3: Code for Support Vector Machines (SVM)


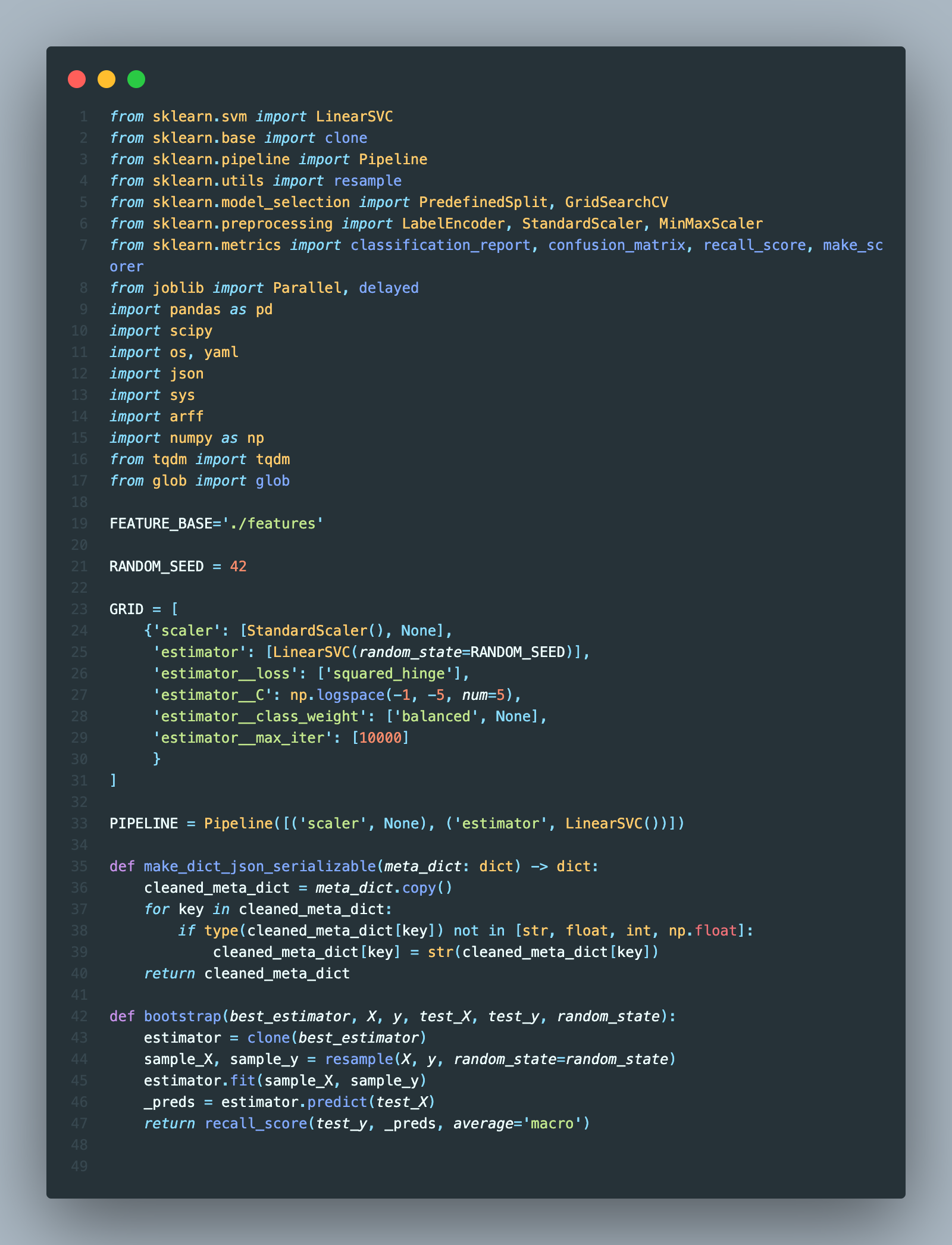


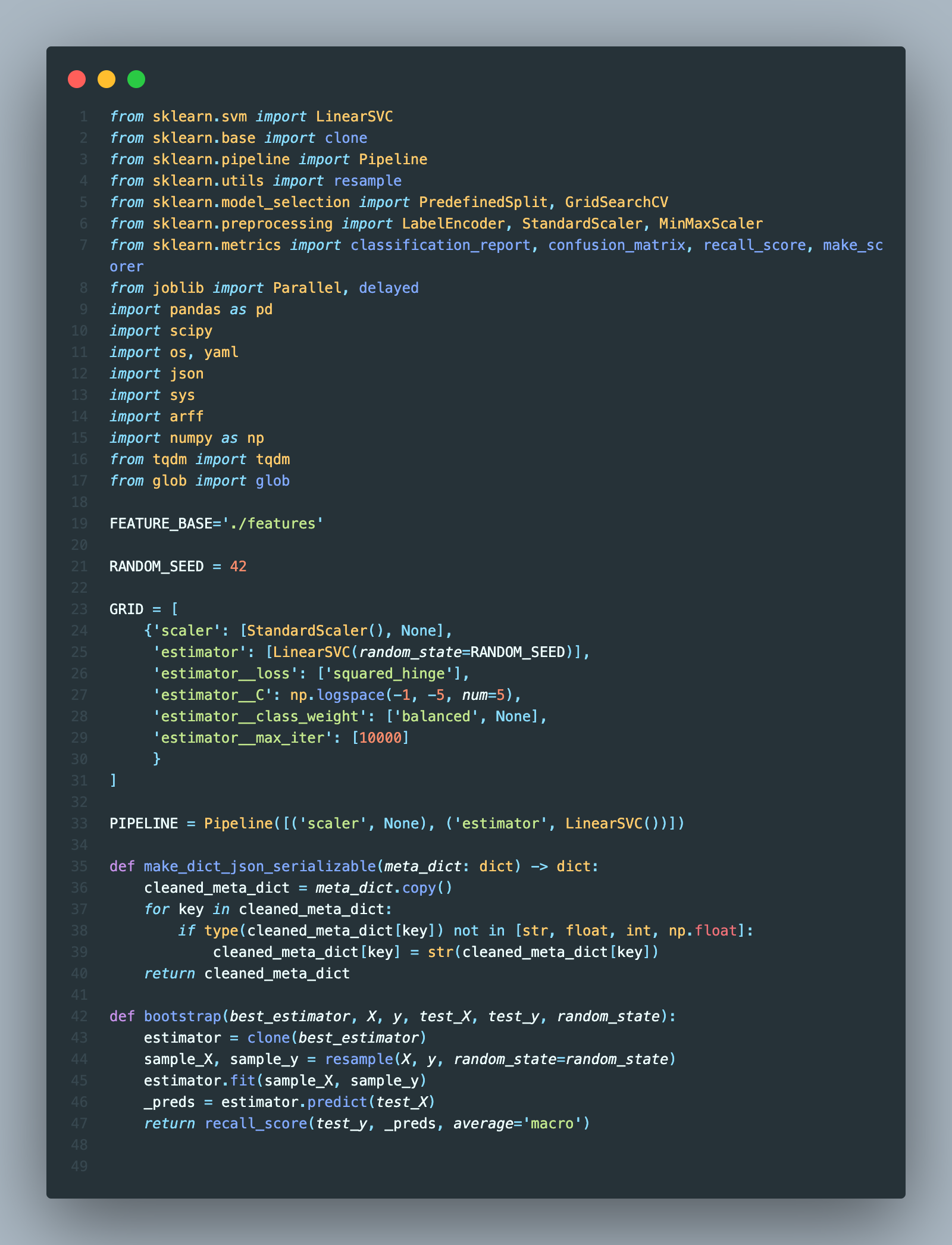


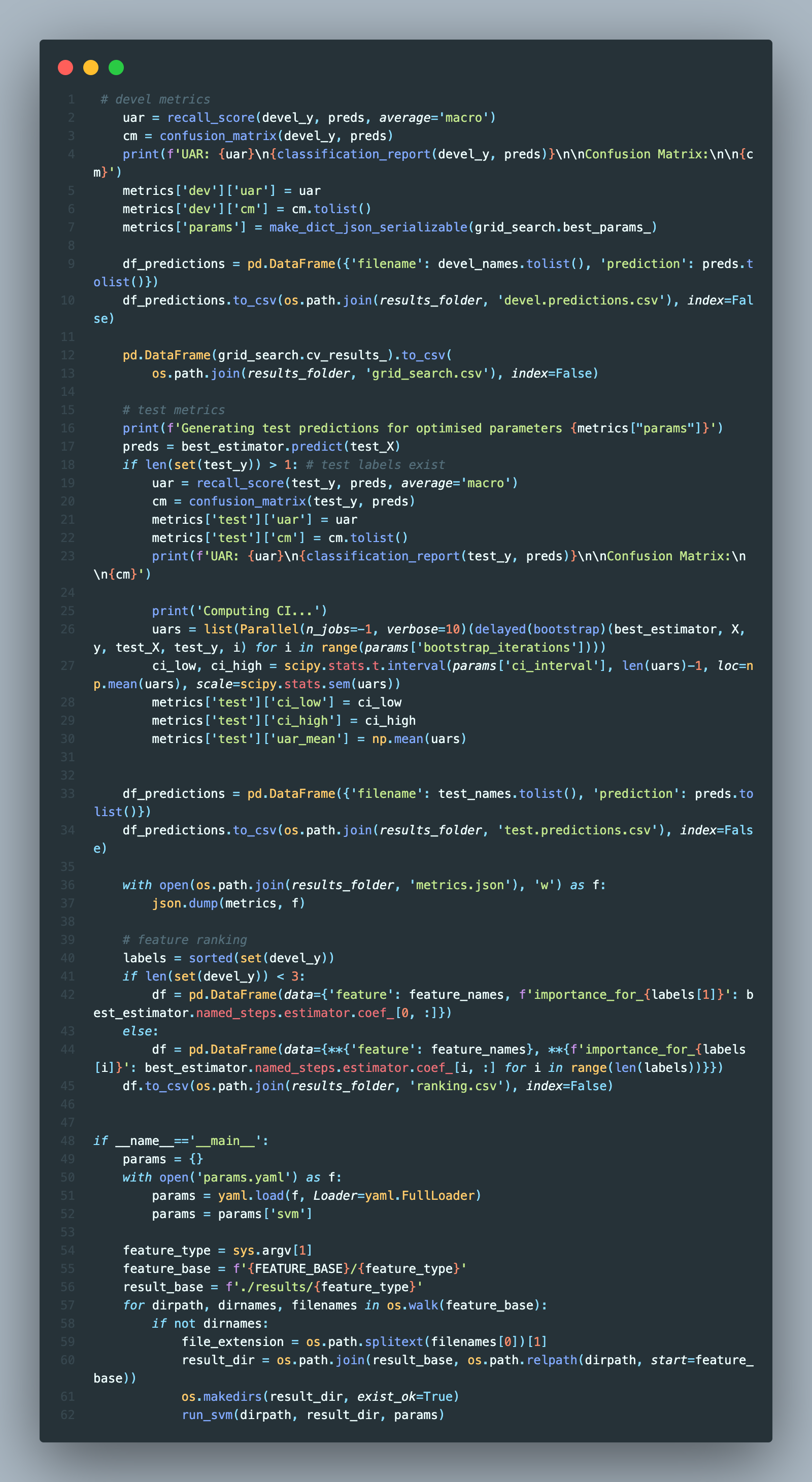


# Supplementary 4: Ethical approval and consent Form


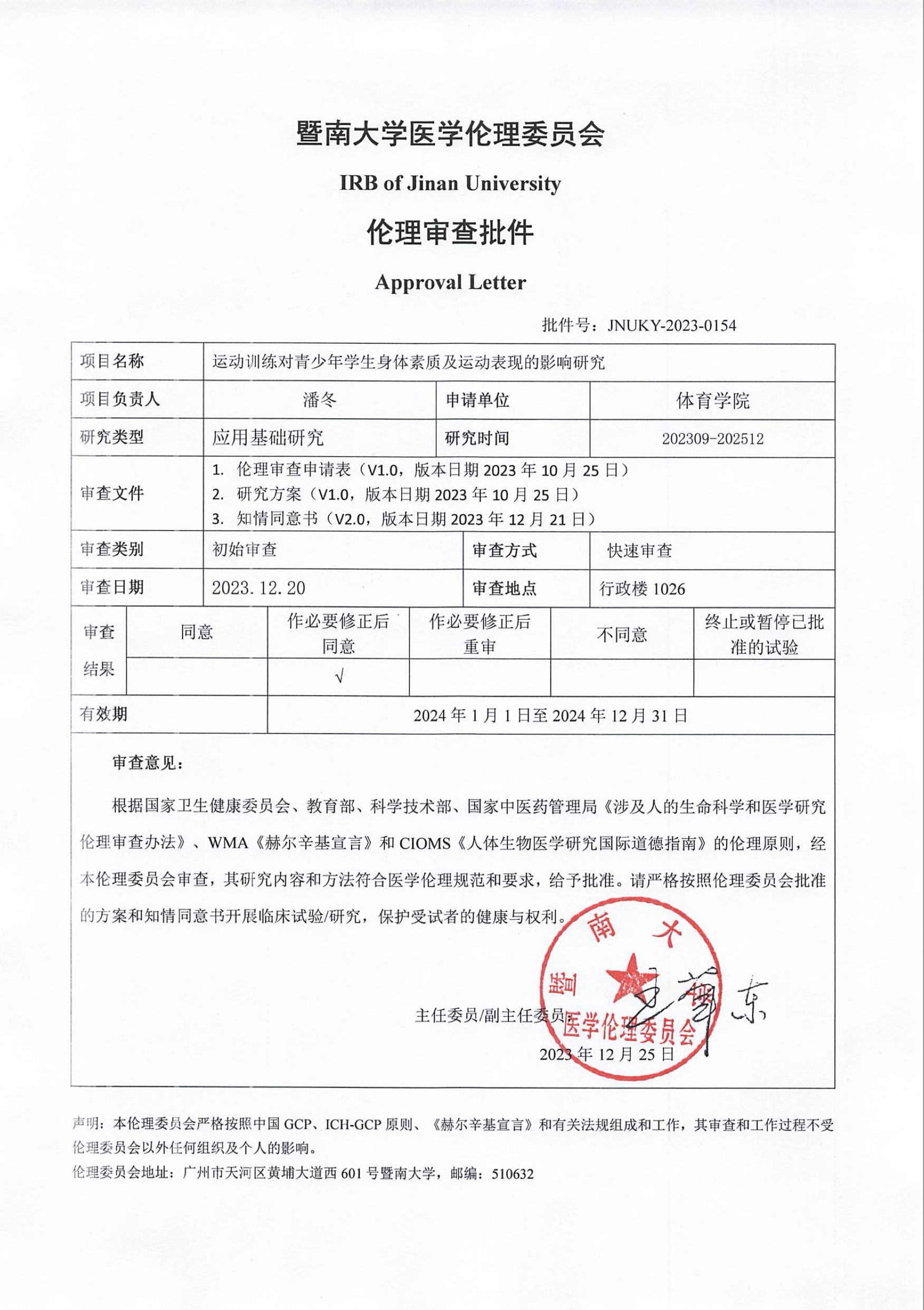

Supplement: Supplementary file 1 [file DataSheet1.docx]
